# Revealing the Ground Deformation and Its Mechanism in the Heihe River Basin by Interferometric Synthetic Aperture Radar and Optical Images

**DOI:** 10.3390/s24154868

**Published:** 2024-07-26

**Authors:** Qunpeng Cui, Yuedong Wang, Pengkun Wang, Ke Tan, Guangcai Feng

**Affiliations:** 1 School of Land Science and Technology, China University of Geosciences, Beijing 100083, China; 1012211216@email.cugb.edu.cn (Q.C.); 1012214122@email.cugb.edu.cn (P.W.); 1012211221@email.cugb.edu.cn (K.T.); 2 School of Geosciences and Info-Physics, Central South University, Changsha 410083, China; fredgps@csu.edu.cn

**Keywords:** Heihe River Basin, deformation monitoring, InSAR, integrated remote sensing, inland river basin

## Abstract

The Heihe River Basin (HRB), located on the northeast margin of the Qilian Mountains, is China’s second largest inland river basin. It is a typical oasis-type agricultural area in northwest China’s arid and semiarid areas. It is important to monitor and investigate the spatiotemporal distribution characteristics and mechanisms of surface deformation in HRB for the ecology of inland river basins. In recent years, research on HRB has mainly focused on hydrology, meteorology, geology, or biology. Few studies have conducted wide-area monitoring and mechanism analysis of the surface stability of HRB. In this study, an improved interferometric point target analysis InSAR (IPTA-InSAR) technique is used to process 101 Sentinel-1 SAR images from two adjacent track frames covering the HRB from 2019 to 2020. The wide-area deformation of the HRB is obtained first for this period. The results show that most of the surface around the HRB is relatively stable. There are six areas with an extensive deformation range and magnitude in the plain oasis area. The maximum deformation rate is more than 50 mm/year. The maximum seasonal subsidence and uplift along the satellites’ line-of-sight (LOS) direction can be up to −70 mm and 60 mm, respectively. Moreover, we use the Google Earth Engine platform to process the multisource optical images and analyze the deformation areas. The remote sensing indicators of the deformation areas, such as the normalized difference vegetation index (NDVI), soil moisture (SMMI), and precipitation, are obtained during the InSAR monitoring period. We combine these integrated remote sensing results with soil type and precipitation to analyze the surface deformations of the HRB. The spatiotemporal relationships between soil moisture, vegetation cover, and surface deformation of the HRB are revealed. The results will provide data support and reference for the healthy and sustainable development of the inland river basin economic zone.

## 1. Introduction

Heihe River Basin (HRB) is the second largest inland river basin and one of the essential parts of the Hexi Corridor in northwest China’s arid and semiarid areas. HRB has a complete water-ecological-economic complex system [1]. In recent years, with the implementation of the “Digital Heihe” and “Heihe Integrated Remote Sensing Joint Experiment”, several scholars have conducted in-depth research on the HRB by using a variety of technologies, such as active/passive remote sensing carried out by ground, space, satellite, surface hydrological, meteorological, and ecological observation, achieving a series of achievements [1,2,3,4,5,6,7]. The integrated remote sensing monitoring of geohydrology and ecology in the Heihe River Basin can help to better understand the development and health status of inland river basins, which has important research significance and economic value.

Interferometric synthetic aperture radar (InSAR) technology can monitor ground deformation with high spatial resolution and has achieved a series of achievements in earthquakes, volcanic eruptions, urban infrastructure safety, mining subsidence, and groundwater overdraft [8,9,10,11,12,13]. In recent years, InSAR has achieved some outstanding results around HRB. Zhang et al. used high-resolution TerraSAR-X/TanDEM-X data and InSAR technology to derive the annual average change in the elevation of the No. 12 glacier of the Tiger Gulch from 2000 to 2014, which proved that utilizing the TerraSAR-X/TanDEM-X data and differential InSAR (DInSAR) technique to monitor the glacier elevation changes in the Qilian Mountains has good feasibility [14]. Chen et al. utilized 17 ALOS PALSAR images and the Persistent Scatterer InSAR (PS-InSAR) technique to investigate the Qilian Mountains’ Oboling permafrost subsidence, demonstrating the potential of using PSI to study the permafrost thawing process and to assess its impacts on the Tibetan Plateau and vast areas of the Arctic [15]. Chen et al. monitored the temporal deformation of the permafrost zone in the upper reaches of the Heihe River from 2014 to 2016 using Sentinel-1 data [16]. They utilized geothermal temperature data and the Stefan model to invert the amplitude seasonal deformation in the region. Geomorphology, soil type, and active layer thickness have affected permafrost deformation. Peng et al. monitored surface deformation in the perennial permafrost zone of the HRB using Sentinel-1 data based on temperature and soil moisture and estimated the active layer thickness [17]. Yang et al. monitored the three-dimensional (3D) surface deformation in the Jinchuan mining area of HRB by InSAR [18]. The existing studies on HRB mainly focus on using InSAR to monitor the ground deformation in the upper glacial tundra area. There are few reports on the monitoring and investigation of surface stability in the plain oasis area of HRB.

Optical remote sensing (O-RS) can intuitively obtain ecological- and environmental-health-related phenological indices in oasis agricultural areas and is widely used in meteorological, biological, geological, and human activity studies. Many scholars have modeled the hydrological model of the HRB using observatory measured data, multiscale remote sensing data, policy combinations, and human activities [1,2]. Li et al. established a hydrological cycle model of the HRB, generated model-forcing data on the atmosphere, and created a new land cover map [5]. Yang et al. fused hyperspectral images and LiDAR data to conduct a fine crop classification study of the Zhangye Oasis agricultural area in the middle reaches of the Heihe River [19]. Zhong used LiDAR, RTK, UAV, and InSAR data to monitor the surface deformation of thermally thawed karst landscapes in the northern Tibetan Plateau [20]. They demonstrated that temperature variations and precipitation extremes have an essential influence on the deformation process. Wang et al. analyzed and calculated the distribution of carbon fluxes in the HRB and its climatic factors [7]. Due to irrigation and groundwater recharge, they found significant differences in precipitation effects and soil moisture between the arid oases in northwest China and those outside. Liang et al. used high-spatial-resolution UAV images to conduct thermal thaw-slip monitoring experiments in the Oboling Pass’s permafrost area in the Heihe River’s upper reaches [21]. They extracted a high-precision boundary of thermal thaw-slip in permafrost. Ruehr et al. proved that the oasis effect in the HRB is weakened due to the influence of irrigation by IBPM theory [6]. Moreover, some studies have analyzed and evaluated the ecological and environmental quality of HRB by using the indices obtained by O-RS, such as the humidity index, desertification index, normalized water body index, water use efficiency, surface temperature, and leaf area index [4,22,23]. Existing studies for the plain oasis area of HRB are mainly based on O-RS. There are few studies on surface stability and ecological health assessment in the plain oasis area of HRB.

In this study, we combined InSAR and O-RS to conduct a comprehensive observation and analysis of the ground stability of HRB. We collected the Sentinel-1 dataset and corresponding MODIS images covering the HRB during 2019–2020. The InSAR data were processed using an advanced time-series InSAR (TS-InSAR) technique to obtain the surface deformation in the plain oasis area of the HRB. The O-RS data were processed using the Google Earth Engine platform to extract remote sensing indicators such as the surface normalized difference vegetation index (NDVI), soil moisture (SMMI), and precipitation in the HRB. Finally, combined with the local geohydrological data, we carried out an integrated remote sensing analysis of surface stability to reveal the spatiotemporal evolution characteristics and response rules of the surface in the study area.

## 2. Study Area and Datasets

### 2.1. Study Area

**Figure 1:**
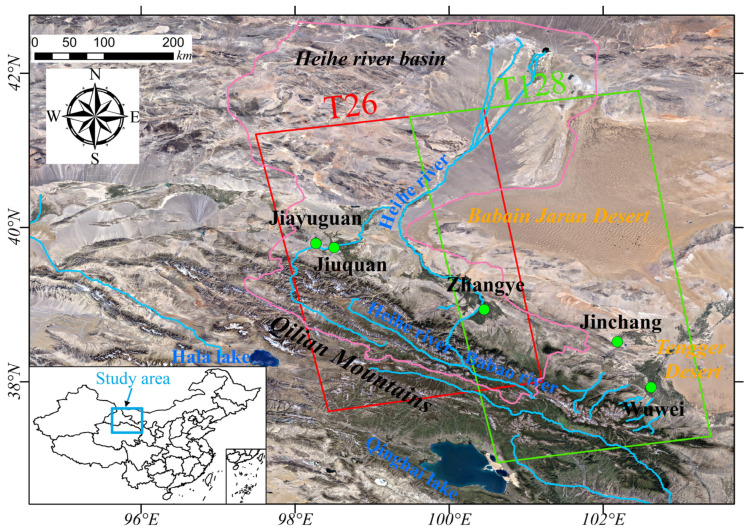
The Heihe River basin. The pink line shows the HRB range. The light blue line shows the approximate spatial distribution of surface runoff. The red and green rectangles are the spatial coverages of the adjacent Sentinel-1 tracks.

The Qilian Mountains are located in the northeastern part of the Qinghai–Tibetan Plateau and the southern edge of the Hexi Corridor, which is the choke point of the Silk Road Economic Belt and nurtures the water sources of six watersheds in China [24]. The HRB, located on the northeast margin of the Qilian Mountains, is a typical basin-type oasis economic zone in arid and semiarid areas in northwest China and is the second largest inland river basin in China (Figure 1). HRB is a multifaceted landscape structure mainly characterized by mountain glacier meltwater and rainfall, oases, and deserts linked by the water cycle [25]. HRB is bordered by the Tengger and Badanjilin Deserts in the east and northeast, respectively, and surrounded by the Gobi in the north and northwest. Its water resources mainly derive from glacial meltwater and rainfall in the Qilian Mountains. The upper reaches of the HRB are the structural uplift area of the Qilian Mountains. The terrain in the middle generally decreases from southeast to northwest and then increases slightly to the Jinta Basin in the north. The downstream reaches the Ejina Banner at the end of the Heihe River and is depressed in the slow uplift zone [22]. The climate of the HRB has apparent variability in the east–west and north–south directions. The average annual temperature is generally higher in the north and lower in the south. The annual precipitation is high in the south and low in the north. The annual evaporation is higher in the northwest and lower in the southeast.

The spatiotemporal distribution of surface water in HRB depends mainly on snow, ice melt, and rainfall in the Qilian Mountains. The intra-annual variability is cyclical. The dry season is winter and spring (October to March). From April to June, ice and snow melt accelerates with the temperature rise. This period also marks the peak of spring irrigation in the middle reaches of the basin. So, the surface runoff drops to a low value and even cuts off. The rainy season is mainly concentrated in July to September. During this period, precipitation and glacial meltwater increase. The surface runoff exceeds 55% of the total annual runoff. During September, large amounts of irrigation return water and groundwater overflow from the cropping areas, creating a peak in surface runoff. With the winter irrigation of planting areas in October and the decrease in precipitation, the runoff volume decreases again and reaches its lowest value in November. Population distribution, human activities, and the spatial distribution of planting areas in HRB are correlated with water resources. A developed pastoral and tourism economy characterizes the upper reaches of HRB. Irrigated agriculture in the middle reaches of HRB is highly developed. A proportion of 60% of the arable land in the basin is concentrated in this area, with corn and wheat as the main cash crops. The lower reaches are characterized by desert areas, with desert pastoralism being the mainstay [22].

### 2.2. Datasets

#### 2.2.1. InSAR Data

In this study, we acquired 101 Sentinel-1 SAR images from the adjacent tracks covering the major oasis areas of the HRB from January 2019 to December 2020 to monitor the wide-area ground deformation. Figure 1 shows the image coverages. The image information is shown in Table 1.

#### 2.2.2. Optical Remote Sensing Data

(1) Land cover data

The land cover data came from Esri’s dataset (land use/land cover time series of the world from Sentinel-2) [26]. They are generated using the Impact Observatory’s deep learning AI land classification model. The spatial resolution is 10 m. The temporal resolution is one year. We selected a land cover image of the HRB in 2019.

(2) NDVI data

The NDVI data came from the land vegetation product of NASA (MODIS/061/MOD13Q1) [27], with a spatial resolution of 250 m. The best value was selected from a 16-day image as a pixel value. We obtained the data on HRB from 46 images, one per month, from January 2019 to December 2020.

(3) Soil moisture data

We adopted the 1 km high-quality soil moisture dataset in China released by the A Big Earth Data Platform for Three Poles in 2022 [28,29]. The dataset is based on observing ten soil moisture layers at 1648 stations provided by the China Meteorological Administration. ERA5 land meteorological forcing data, leaf area index, land types, DEM, and soil properties were used as covariates and obtained by machine learning. The data unit is 0.001 m^3^/m^3^. The temporal resolution is one day. The spatial resolution is about 1 km. Data for 2019 and 2020 were used in this study.

(4) Precipitation data

The precipitation data came from A Big Earth Data Platform for Three Poles, which is generated in China through the Delta spatial downscaling scheme based on the global 0.5° climate dataset released by CRU and the global high-resolution climate dataset released by WorldClim [30,31]. The spatial resolution is 0.0083333°, about 1 km. The 24 images obtained in this study represented one image per month for two years.

(5) Soil type data

The soil type data used in this study came from the Institute of Soil Science, Chinese Academy of Sciences [32]. The digital soil type data were compiled digitally based on the original paper soil map, with a scale of 1:400,000.

## 3. Methodology and Data Processing

### 3.1. InSAR Data Processing

#### 3.1.1. Monitoring the Ground Deformation Time Series

**Figure 2:**
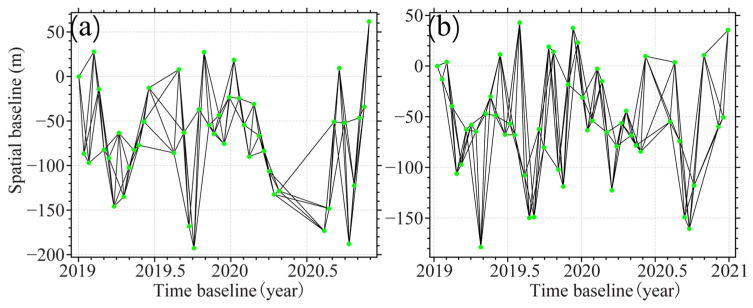
Small baseline set interference pair combination for T128 (**a**) and T26 (**b**). The green dots identify the spatio-temporal acquisition information for each image.

The acquired adjacent tracks of the Sentinel-1 dataset were first registered and resampled to the same coordinate frame, respectively. Then, the SAR data from the same track were composed into multitemporal InSAR pairs by setting temporal and spatial baseline thresholds based on the idea of small baseline sets [33]. Table 1 shows the number of these pairs. Figure 2 shows the network configuration of these pairs. 

For one of the differential interferograms generated from the SAR images acquired at the moment of $t_{A}$ and $t_{B}$ ($t_{A}$ > $t_{B}$), the interferometric phases δϕ can be written as

(1) $$\begin{array}{c} \delta \phi =\phi_{B}-\phi_{A}\\ \approx \frac{4\pi }{\lambda }[d(t_{B})-d(t_{A})]+\Delta \phi_{topo}+\Delta \phi_{APS}(t_{B},t_{A})+\Delta \phi_{noise} \end{array}$$

where λ is the radar wavelength. $d(t_{B})$ and $d(t_{A})$ are the ground deformations at the times of $t_{B}$ and $t_{A}$. $\Delta \phi_{topo}$ is the residual topographic phase. $\Delta \phi_{APS}(t_{B},t_{A})$ is the atmospheric delay phase. $\Delta \phi_{noise}$ denotes the decoherent noise.

In data processing, we used the Shuttle Radar Topography Mission (SRTM) DEM [34] and the GAMMA software (v20231211) [35] to process the multitemporal InSAR pairs. A multilook factor of 20 × 4 (distance × azimuth) was selected. An improved IPTA-InSAR method was adopted to process the dataset of each frame to obtain the deformation time series [36,37]. A topographic-dependent atmospheric delay error correction strategy was adopted to suppress the local atmospheric delay errors [38]. Finally, we obtained the time-series deformation of each frame. The final resolution of the monitoring results after geocoding was about 56 m in the east–west and north–south directions.

#### 3.1.2. Wide-Area InSAR Results Splicing

**Figure 3:**
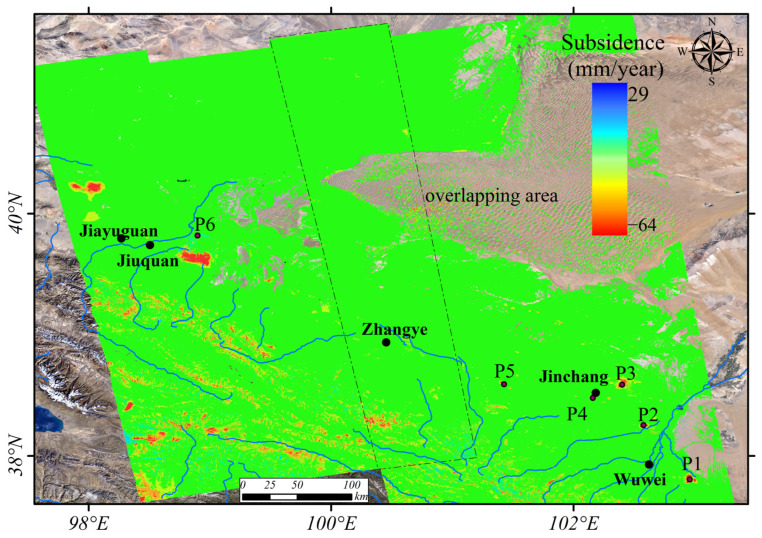
The subsidence rate results of HRB from 2019 to 2020. The black dotted line area which is the overlapping area of the two tracks represents the selected accuracy verification area in Section 4.3.

To obtain the wide-area deformation results with a consistent datum, we adopted a multi-adjacent tracks deformation splicing method to fuse the obtained adjacent track deformation results [39]. To suppress the inconsistency in observation geometries between the results from the adjacent tracks, the LOS deformation ($d_{LOS}$) of the two frames was converted to the vertical direction ($d_{U}$),

(2) $$d_{LOS}=cos\theta \cdot d_{U}$$

where θ is the radar incidence angle, based on the repeated observations in the overlapping area of the adjacent tracks, and the reference deviations of the two tracks were corrected. Then, we obtained the wide-area deformation of HRB (Figure 3). Since this study mainly focused on the surface deformation in the plain oasis area of the HRB that is closely related to human activities, the deformation in the Qilian Mountain area was not discussed and analyzed in the follow-up analysis.

### 3.2. Optical Remote Sensing Data Processing

The NDVI can reflect the vegetation coverage. The calculation formula of NDVI is [40]

(3) NDVI=(NIR−R)/(NIR+R)

where NIR is the near-infrared band’s reflection value. R is the reflection value of the red band.

We obtained the surface NDVI data in July 2019, January 2020, and July 2020 based on O-RS. Subsequently, we calculated the NDVI differences for each pixel among those of July 2020, July 2019, and January 2020 to assess interannual and seasonal variations in NDVI.

The impact of large-scale irrigation in agricultural areas on soil moisture mainly affects the shallow surface, as surface water rarely infiltrates deep into the soil [3]. Therefore, we selected the topsoil moisture data at a depth of 10 cm from July 2019, January 2020, and July 2020 to reflect the impact of human activities. We compared the soil moisture differences in July 2020, July 2019, and January 2020 to evaluate the spatiotemporal variations in soil moisture in the HRB.

### 3.3. Correlation Analysis of O-RS and InSAR Deformation

We used the ArcMap platform to conduct an integrated remote sensing analysis of the plain oasis area in the HRB. A deformation rate threshold of 10 mm/year was selected to screen the regions with significant deformation. We set the DEM and NDVI ranges to remove the deformation points in the upstream mountain and downstream desert regions and obtain the significant deformation point data in the middle reaches. 

Geographically weighted regression (GWR) is a spatial statistical technique that mitigates the influence of spatial heterogeneity on regression models. It can provide a better fit for linear regression in local areas [41]. In this study, we used the deformation rate as the dependent variable and the differences in NDVI and soil moisture between 2019 and 2020 as the independent variables. We applied GWR to conduct the multiple regression analysis,

(4) $$y_{i}=\beta_{i0}+\sum_{k=1}^{p}\beta_{ik}x_{ik}+\varepsilon_{ir}$$

where $y_{i}$ is the dependent variable of position i. $\beta_{i0}$ is the intercept coefficient of position i. $x_{ik}$ is the *k*-th explanatory variable of position i. $\beta_{ik}$ is the local regression coefficient of the *k*-th explanatory variable of position i. $\varepsilon_{ir}$ is the random error term associated with position i. Note that *i* is usually indexed by two-dimensional geographic coordinates ($u_{i}$, $v_{i}$), indicating the regression point’s location. In this study, we set the bandwidth to 500 m, using data from all deformation points within a radius of 500 m around the central deformation point to perform weighted regression.

We used the local $R^{2}$ ($R_{local}^{2}$) to validate the precision of the GWR model at each spatial location [41]. It was calculated using the following formula:

(5) $$R_{local}^{2}=\sum_{i=1}^{n}{\left(y_{i}-{\hat{y}}_{i}\right)}^{2}/\sum_{i=1}^{n}{\left(y_{i}-\bar{y}\right)}^{2}$$

where $y_{i}$ is the i-th observation of the dependent variable. ${\hat{y}}_{i}$ is the *i*-th predicted value of the model for the dependent variable. $\bar{y}$ is the global mean of the dependent variable. n is the number of observations. All $R_{local}^{2}$ are weighted to obtain global $R^{2}$. $R^{2}$ takes the value 0–1. The closer it is to 1, the better the model fits. 

We randomly selected a point in the agricultural planting area with significant deformation to analyze the correlation between the remote sensing index and deformation rate in the time dimension. We took the radius circle of 2000 m around the selected point as the research area. We used Google Earth Engine (GEE) and MATLAB to obtain the NDVI time series and SMMI for these significant deformation areas during the deformation period. These time series were then compared with the InSAR deformation time series.

We conducted Pearson correlation analysis on the smoothed deformation, SMMI, and NDVI. The correlation coefficient in the Pearson correlation analysis can be expressed as follows [40]:

(6) $$R=\sum (X_{i}-\bar{X})(Y_{i}-\bar{Y})/\sqrt{\sum {\left(X_{i}-\bar{X}\right)}^{2}\sum {\left(Y_{i}-\bar{Y}\right)}^{2}}$$

where $X_{i}$ and $Y_{i}$ are the i-th observations of the two variables. $\bar{X}$ and $\bar{Y}$ are the average values of X and Y, respectively. Pearson’s R is measured in correlation coefficients: 0 indicates no linear relationship between variables, whereas 1 indicates an ideally increasing linear relationship and vice versa.

It is crucial to conduct regression analysis to examine the statistical relationship between long-term change trends in multiple variables [40]. In this study, we considered the deformation rate as the dependent variable and NDVI and SMMI as the independent variables for linear regression analysis. The time-series NDVI and SMMI were the independent variable x, and the deformation rate was the dependent variable y:

(7) $$y=\alpha +kx_{i}+\varepsilon_{i}$$

where α is the coefficient. k is the slope of the linear regression. $\varepsilon_{i}$ are the random errors. We used each set of corresponding independent variables (NDVI or SMMI) and the dependent variable deformation rate ($x_{i}$, $y_{i}$) (*i* = 1, 2 …*n*) to determine the slope parameter k:

(8) $$k=\sum_{i=1}^{n}(x_{i}-\bar{x})(y_{i}-\bar{y})/\sum_{i=1}^{n}{\left(x_{i}-\bar{x}\right)}^{2}$$

where $\bar{x}=\frac{1}{n}\sum_{i=1}^{n}x_{i}$ and $\bar{y}=\frac{1}{n}\sum_{i=1}^{n}y_{i}$.

Finally, we used the root-mean-square error (RMSE) to validate the regression model. The smaller the RMSE, the better the model accuracy.

(9) $$RMSE=\sqrt{\frac{1}{n}\sum_{i=1}^{n}{\left(y_{i}-{\hat{y}}_{i}\right)}^{2}}$$

where n is the number of samples. $y_{i}$ is the *i*-th actual value. ${\hat{y}}_{i}$ is the *i*-th predicted value.

## 4. Results

### 4.1. Surface Deformation in HRB

Figure 3 shows the deformation results in HRB. The surface in most areas of the middle and lower reaches of HRB has remained relatively stable, especially around Zhangye City and its northwestern region, with deformation rates below 10 mm/year. Significant deformation areas correlate with human activities such as agriculture and mining. The large-deformation distribution regions are mainly in the northwest of Jiayuguan City (the maximum deformation magnitude exceeds −30 mm/year); the southeast of Jiuquan City (the maximum deformation magnitude exceeds −50 mm/year); the southeast of Wuwei City (P1, the maximum deformation magnitude exceeds −30 mm/year); and the open-pit mining area near Jinchuan National Mining Park, Jinchang City (P4, the maximum deformation magnitude exceeds −50 mm/year). In the farming areas to the southeast and north of Jinchang City (P2, P3), the maximum deformation magnitude reaches −20 mm/year. The Huacaotan Coal Mine and Dongshuiquan Coal Mine are located in Laojun Township, Shandan County, Zhangye City (P5, the maximum deformation magnitude exceeds −20 mm/year).

### 4.2. O-RS Results

**Figure 4:**
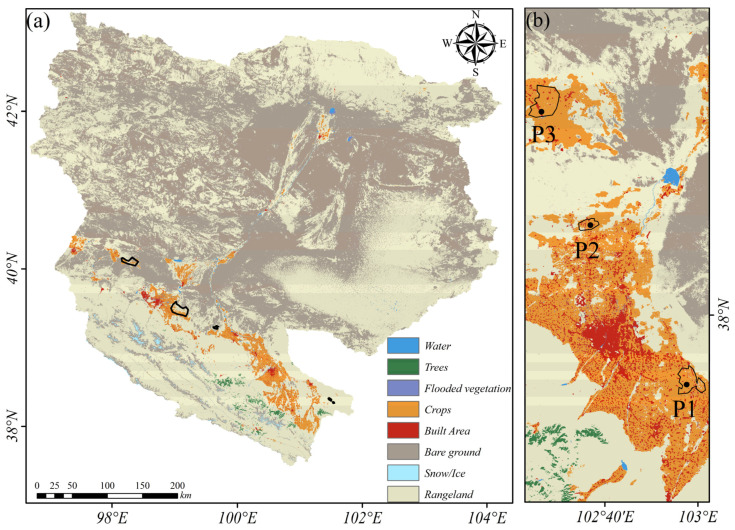
(**a**) The overall land cover map of HRB. (**b**) The land cover map of the significant deformation areas, with the black line indicating the range of the significant deformation areas obtained in Figure 3. P1, P2, and P3 are significant deformation areas located in agricultural planting areas.

Figure 4 shows the land cover types in the HRB. We inquired about the location map of significant deformation areas in the midstream of HRB obtained in Figure 3 in the land cover map. We found that surface deformation mainly occurs in the irrigated planting areas, mining areas, and saline–alkali regions. Among them, P1, P2, and P3 are irrigated agricultural areas. P4 and P5 are mining areas. P6 is barren land.

**Figure 5:**
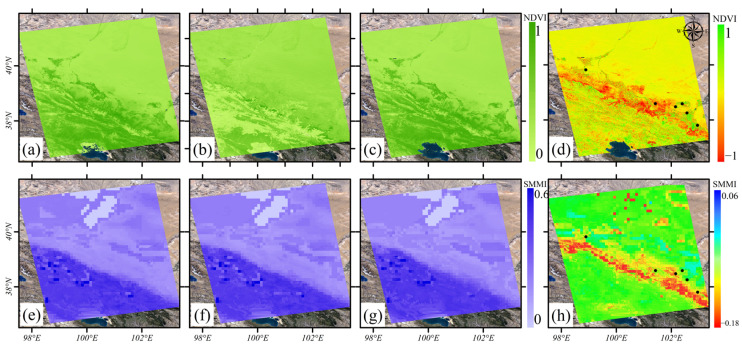
(**a**,**e**) NDVI and soil moisture in summer 2019. (**b**,**f**) NDVI and soil moisture in winter 2020. (**c**,**g**) NDVI and soil moisture in summer 2020. (**d**,**h**) The difference between NDVI and soil moisture in the summer of 2020.

**Figure 6:**
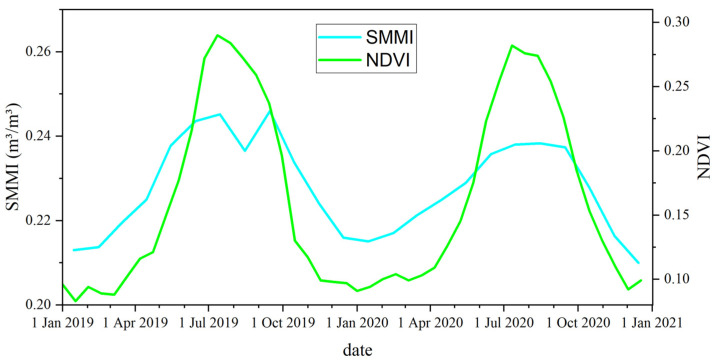
Temporal variation in NDVI and soil moisture in HRB.

**Figure 7:**
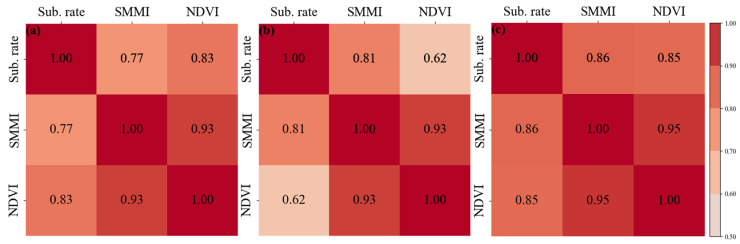
(**a**–**c**) Thermal maps of the correlation coefficient between subsidence rate, SMMI, and NDVI at P1, P2, and P3, respectively. The redder the color, the higher the correlation.

Figure 5 shows the NDVI and soil moisture distribution and their changes in the HRB. The upstream, midstream, and downstream regions indicate significant spatial differences in NDVI and soil moisture. The overall NDVI in the basin is low in winter. However, in summer, the NDVI exceeds 0.7 in most upstream areas and near rivers and irrigation channels in the midstream. Meanwhile, the NDVI in other areas and the downstream region generally have values below 0.2. Soil moisture decreases from the upper reaches to the lower reaches. Vegetation cover, soil moisture, and precipitation show significant seasonal and interannual changes. The NDVI and soil moisture in the midstream region exhibit substantial interannual variability. The above parameters show a trend of being higher in summer and lower in winter (Figure 6). Table 2 presents the mean changes in NDVI and soil moisture in HRB. The seasonal variation in NDVI is about 0.2. The variation in soil moisture is about 0.02. Compared to 2019, NDVI and soil moisture in the significant deformation areas within the irrigation zone increased in 2020. We conducted a GWR analysis on the differences in NDVI, soil moisture, and the deformation rate between the two years. The global coefficient of determination $R^{2}$ = 0.9. Moreover, we analyzed the correlation between the pairs of time-series NDVI, soil moisture, and subsidence rate. Figure 7 shows the heatmap of the correlation among these variables. There is a significant positive correlation, with the correlation between NDVI and soil moisture exceeding 0.9. The correlation between subsidence rate, soil moisture, and NDVI ranges from 0.7 to 0.9. These results indicate a close spatial correlation between NDVI, soil moisture, and subsidence in the significant deformation areas of HRB.

### 4.3. Reliability Validation

(1) InSAR

**Figure 8:**
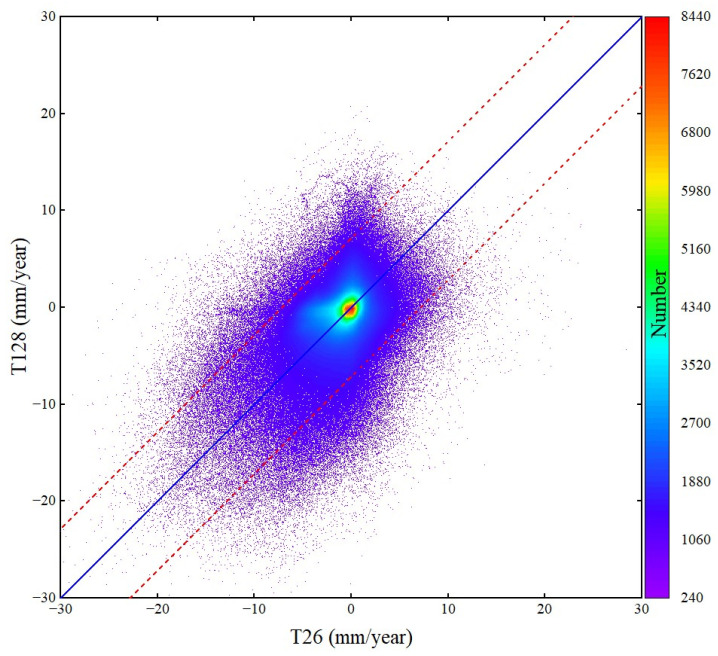
Comparison between the results obtained by the adjacent track data of T26 and T128. The red dotted lines denote the value that is three times the root-mean-square error.

As shown in Figure 3, the wide-area deformation of HRB from the Sentinel-1 data of adjacent tracks has a good spatial distribution and magnitude consistency. To quantitatively assess the reliability of the deformation results, we compare the average subsidence rates extracted from the overlapped areas of the two adjacent InSAR tracks, i.e., the black dotted line area in Figure 3. The result is shown in Figure 8. The RMSE between the deformation results of T26 and T128 is 2.8 mm. The differences at most points are smaller than three times the RMSE (between the red dotted lines in Figure 8). The good consistency and minor errors between the independent solution results of the adjacent track prove the reliability of the InSAR results.

(2) Optical remote sensing

**Figure 9:**
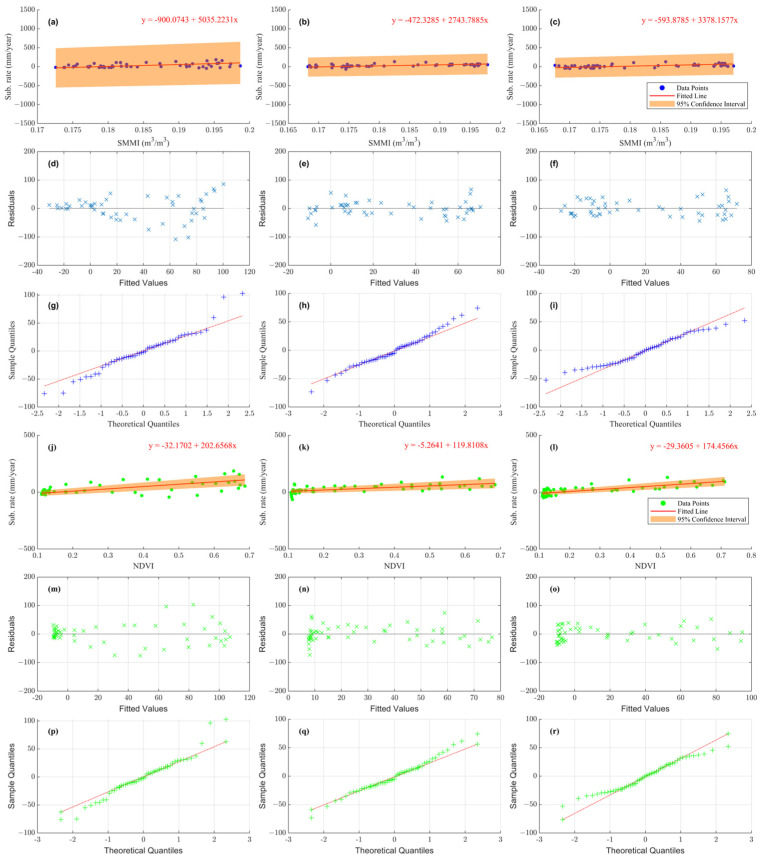
(**a**–**c**) The regression of subsidence rate to soil moisture in significant subsidence regions around P1, P2, and P3. (**d**–**f**) The soil moisture regression residuals. The horizontal axis represents the deformation rate values predicted by SMMI, while the vertical axis represents the difference between predicted and actual deformation rate values. (**g**–**i**) QQ plots of the soil moisture regression residuals. (**j**–**l**) The regression of subsidence rate to NDVI in P1, P2, and P3. (**m**–**o**) The NDVI regression residuals. The horizontal axis represents the deformation rate values predicted by NDVI, while the vertical axis represents the difference between the predicted and actual deformation rate values. (**p**–**r**) QQ plots of the NDVI regression residuals.

For the significant deformation areas in the irrigated planting area of HRB, the soil moisture, NDVI, and ground subsidence show periodic changes. We conducted a linear regression analysis with soil moisture and NDVI as independent variables and ground subsidence as the dependent variable at the significant deformation points in Figure 3. The result is shown in Figure 9. The orange areas in Figure 9 represent the confidence intervals of 95%. Most soil moisture and NDVI prediction points fall within the confidence intervals (Figure 9a–c,j–l). The residuals are roughly uniformly distributed around zero (Figure 9d–f,m–o). The Quantile–Quantile (QQ) plots of the residuals (Figure 9g–i,p–r) show that the residuals align well with the theoretical line, indicating a normal distribution. The RMSEs between SMMI and NDVI of P1, P2, and P3 are shown in Table 3. The RMSE is obtained by calculating the mean of the squared differences between the predicted and observed values and then taking the square root of that mean. The error of the regression results is within centimeters, and the error range is small, confirming the reliability of the regression accuracy.

## 5. Discussion

### 5.1. Factors Affecting the Development of Deformation at Spatial Dimensions in HRB

**Figure 10:**
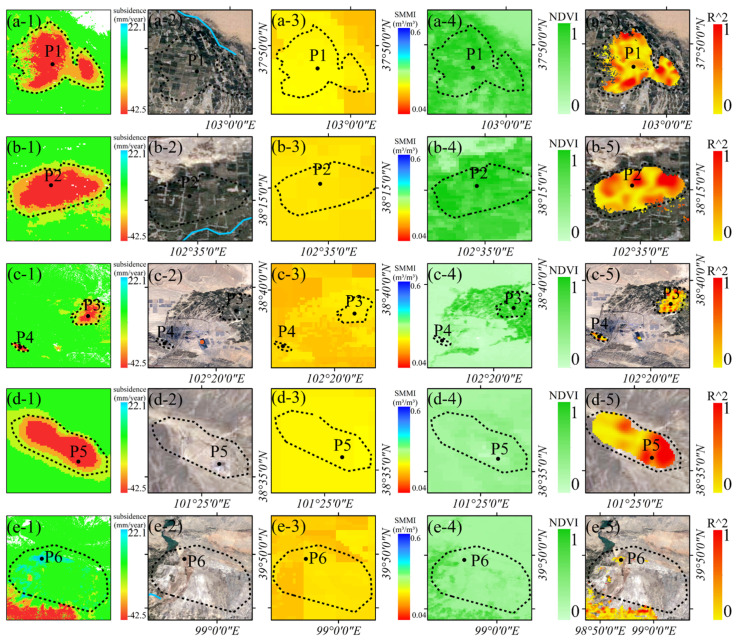
(**a-1**–**e-1**) The ground subsidence in the regions where points P1 to P6 are located. (**a-2**–**e-2**) The optical images of these areas. (**a-3**–**e-3**) The soil moisture levels of these areas in the summer of 2020. (**a-4**–**e-4**) The NDVI of these areas in the summer of 2020. (**a-5**–**e-5**) The multisource regression local subsidence coefficients $R^{2}$ for the inter-annual differences in soil moisture and NDVI in these areas.

HRB has dozens of deformation concentration areas (Figure 3). According to different deformation induction mechanisms, these deformations can be categorized into three types: (1) seasonal deformation caused by groundwater extraction for agricultural irrigation; (2) ground subsidence caused by mining; and (3) local ground deformation caused by seasonal precipitation. We selected several representative deformation areas for integrated remote sensing analysis (Figure 10).

In HRB, population, production, and living agglomerations are highly correlated with surface runoff, groundwater distribution, and recharge. Water use in Heihe River’s middle and lower reaches mainly relies on implementing water diversion policy and water transfer projects [25]. Surface runoff makes it difficult to meet the local water demand. Irrigation water in agricultural areas is in high demand for groundwater. The local groundwater replenishment sources include Qilian Mountain ice and snow meltwater, surface runoff and infiltration, and agricultural irrigation infiltration. Abundant recharge sources help with the rapid replenishment of aquifers. Therefore, the surface is generally stable.

Points P1, P2, and P3 are in the agricultural cultivation area (Figure 10a–c). Local subsidence rates near the three points exceed −20 mm/year. We jointly analyzed the surface subsidence, the soil moisture, and the NDVI difference. The mean values of soil moisture and NDVI were higher in the summer of 2020 than in the summer of 2019. The GWR analysis yielded a close correlation among NDVI, soil moisture, and subsidence in spatial distribution. The change in subsidence rate originated from the change in soil moisture and vegetation cover. Groundwater over-extraction caused by agricultural irrigation is highly correlated with surface subsidence.

P4 is located southeast of Jinchuan National Mining Park in Jinchang City (Figure 3). A mining-caused subsidence area is nearby. An open-pit tailings dam exists approximately 10 km east of P4 (within the lower left red point of Figure 10c-2). Subsidence with rates over −10 mm/year exists within the southeast portion of this tailings dam. P5 is located in the Huacaotan coal mining area (Figure 10d). The Dongshuiquan Coal Mine is located about 5 km southeast of the mine, and the surface of the mine also has subsidence. Compared with 2019, soil moisture in mining areas increased in 2020. GWR analysis shows a close correlation between the spatial distribution of soil moisture and subsidence rate. This may be due to the subsidence and destruction of the above strata, which destroys the original geological and hydrological structure and leads to soil moisture changes.

P6 is located in a saline area. The Beida River and the Yuanyangchi Reservoir are near this area. There is a surface uplift near this area, with an uplift rate along the LOS direction of about 13 mm/year. GWR analysis showed that soil moisture and deformation rate correlated highly in spatial distribution. Hence, the local deformation may be due to the seasonal change in the runoff volume of the river and the rise in the reservoir level, which will lead to the expansion of saline–alkali soil and ground uplift in the region.

### 5.2. Factors Affecting the Development of Deformation at Time Dimension in HRB

**Figure 11:**
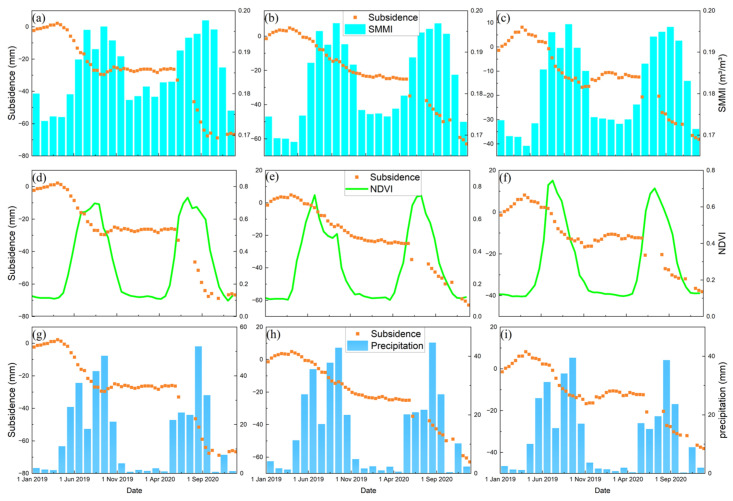
(**a**–**c**) Comparison of the time-series soil moisture (SMMI) and subsidence at points P1, P2, and P3, respectively. (**d**–**f**) Comparison of the time-series NDVI and subsidence at points P1, P2, and P3, respectively. (**g**–**i**) Comparison of the time-series precipitation and subsidence at points P1, P2, and P3, respectively.

**Figure 12:**
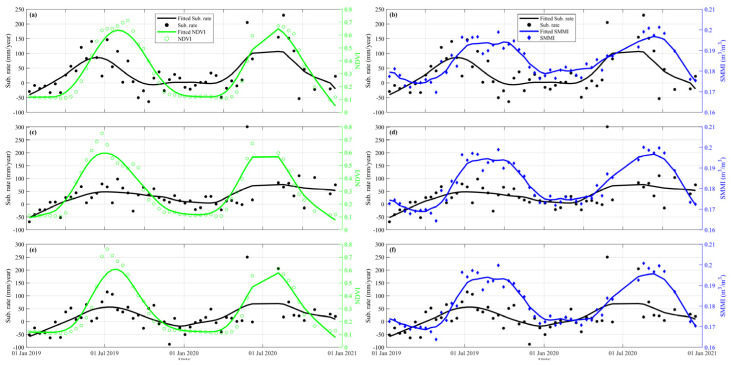
(**a**,**c**,**e**) Comparison of NDVI and subsidence rate in P1, P2, and P3, respectively. (**b,d,f**) Comparison of soil moisture and subsidence rate in P1, P2, and P3, respectively. The scatter represents the original value. The line represents the fitting value.

To analyze the time-dimensional characteristics between surface deformation and its correlations, we randomly selected several characteristic points in the significant deformation areas, as shown by the black dots of P1~P6 in Figure 10. Accelerated subsidence of the surface occurred mainly in spring and summer, with the maximum deformation exceeding −60 mm. The surface deformation slowed down in late summer and early fall. It tends to stabilize or lift in autumn and winter. This phenomenon is mainly due to the accelerated growth of crops in spring and summer when soil moisture is low. Despite the increase in precipitation, the local arid climate and weak precipitation could not meet the demand for agricultural irrigation. We plot the time-series deformation, soil moisture, NDVI, and precipitation of the points located in the agricultural areas (Figure 11). There is a significant correlation between the subsidence time series and the seasonal variations in soil moisture and NDVI. The change in deformation rate is ahead of the change in NDVI. The agricultural areas need to extract lots of groundwater to irrigate the farmland, resulting in significant aquifer loss and accelerated ground subsidence. At the same time, the fast-growing crops caused the local NDVI to rise. The seasonal variation in surface NDVI exceeded 0.7. With the growth of crops and uninterrupted irrigation, the local soil moisture gradually increased. The seasonal variation in soil moisture exceeded 0.03 m^3^/m^3^. As a result, the soil moisture trend is about one month later than subsidence, while the NDVI trend is about half a month later than subsidence (Figure 12).

We derived a significant positive correlation between deformation, soil moisture, and NDVI through correlation calculations. The HRB has an arid climate with low precipitation and high evaporation. Soil moisture rises mainly from rivers, vegetation, and irrigation. Compared with the area covered by surface runoff with sufficient water sources, a large part of the agricultural cultivation area, which is far away from surface runoff and mainly relies on dry/branch/lateral/agricultural canals for water diversion, relies on groundwater pumping for irrigation. In Figure 3, the agricultural areas with large subsidence magnitudes and extents are mainly water-deficient areas, such as around the location of P2 (Figure 10b). The Shiyang River flows in a southwest-to-northeast direction, which is about 13 km southeast of the subsidence area of P2. Although there are dense agricultural fields in the water conveyance line and its vicinity, the surface is relatively stable. The subsidence rate is low. However, there is an apparent large-scale subsidence in the area away from the surface runoff, especially in and around point P2. For large irrigated agricultural cultivation areas, only the surface runoff from distant trunk canals deployed via branch, lateral, or agricultural canals to converge may not meet the water demand of the substantial agricultural production and domestic use. Without adequate surface water, irrigation water will mainly rely on groundwater extraction. The lack of supply sources will lead to overexploitation of local aquifers, resulting in land subsidence.

**Figure 13:**
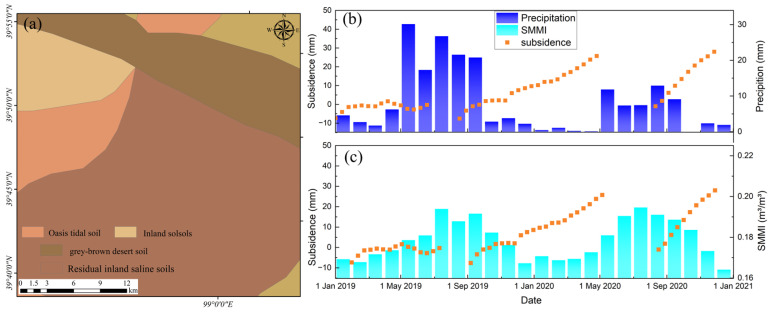
(**a**) The soil type in the deformation area around P6. (**b**) The correlation map between precipitation and deformation. (**c**) The correlation map between soil moisture and deformation.

Point P6 shows a seasonal uplift (Figure 13). Combined with the optical images, the surface cover of the ground uplift area is mainly bare soil gob. Although there is surface runoff in this area, there is a large area of saline soil near the runoff. Severe soil salinization makes it challenging to grow crops. Therefore, the surface of the area tends to stabilize during the season when groundwater extraction is more severe. During the rainy period in August and after, the saline soil layers in the region swell with the replenishment of surface water, causing the surface to lift (Figure 13). The formation of this feature may be related to the high local water table, high salinity, and easy salt accumulation.

### 5.3. The Importance of Integrated Remote Sensing for Inland River Basin Monitoring

Radar remote sensing can obtain high-precision deformation information on the ground surface. Optical remote sensing can intuitively obtain the surface-ecological-health-related indexes. Combined with the two types of remote sensing techniques, integrated remote sensing observation of the ground surface can more comprehensively reveal the surface stability and its influencing factors in different periods and objectively evaluate the local ecological health. In this study, we used integrated remote sensing to monitor the surface stability of HRB. We analyzed the spatiotemporal evolution and triggering mechanisms of surface deformation in the plain oasis area of the HRB by combining soil moisture, NDVI, precipitation information, and optical images. The integrated remote sensing observation will support the economic and social development and ecological environmental protection of the HRB.

Inland river basins in western China are extensive and arid [5]. Many inland river basins around the world share these characteristics. Due to their unique climatic conditions, fragile ecological balance, and significant economic role, an integrated remote sensing survey and analysis of these areas is essential. Integrated remote sensing can provide valuable insights for economic development and ecological, hydrological, and geological assessments of other inland river basins. This study can provide a reference for the application of integrated remote sensing technology in this kind of area. 

## 6. Conclusions

This paper uses an improved IPTA-InSAR technique and 101 SAR images from two adjacent track Sentinel-1 frames to obtain the distribution of surface deformation in the plain oasis area of HRB. The surface is generally stable in HRB, with large-scale deformation primarily occurring in the oasis. The maximum annual average deformation rate exceeds −50 mm/year. The maximum seasonal subsidence and uplift in localized areas reach −70 mm and 60 mm, respectively. We also obtained O-RS data on NDVI, SMMI, and precipitation in HRB. Regression analysis in the significant deformation area revealed a close correlation between surface deformation and both soil moisture and NDVI, with a spatial-dimensional global coefficient of determination $R^{2}$ = 0.9. The correlation coefficient from the time-series analysis is greater than 0.7, indicating a high correlation. The RMSE of the regression is less than 40 mm/year, indicating high precision. The overall trend in soil moisture lagged behind the trend in deformation rate by about one month, while the overall trend in NDVI lagged by about half a month.

This study provides an exploratory analysis of the surface stability of HRB using integrated remote sensing and limited remote sensing data. We have identified the spatiotemporal distribution patterns and characteristics of surface deformation in HRB. This study fills gaps in understanding the spatiotemporal characteristics of the overall deformation in HRB. It also enhances the region’s integrated remote sensing observation technology system. In the future, we will use more sources and extended remote sensing data to conduct continuous monitoring and scientific research in the HRB.

## Figures and Tables

**Table 1:** Parameter information of the used SAR dataset.

Sensor	Sentinel-1	
Track number	T26	T128
Acquisition time	9 January 2019–29 December 2020	2 January 2019–28 November 2020
Image number	47	54
Interference pairs	135	156
Frame number	124, 129	119, 124, 129

**Table 2:** Seasonal and interannual variations in NDVI and soil moisture.

		Seasonal Difference		Interannual Difference	
		NDVI	Soil Moisture (m^3^/m^3^)	Between NDVI	Soil Moisture (m^3^/m^3^)
The overall scope of HRB		0.19	0.02	−0.01	0.01
Southeast of Wuwei City (p1)		0.57	0.02	0.07	0.03
Southeast of Jinchang City (p2)		0.63	0.05	0.12	0.04
North of Jinchang City (p3)		0.55	0.03	−0.001	0.03
Jinchuan Mine Park (p4)		0.04	0.002	−0.03	−0.001
Huacaotan Coal Mine (p5)		0.17	0.03	−0.07	0.03
East of Suzhou District (p)		0.08	0.03	0.0006	0.017

**Table 3:** RMSE of regression between soil moisture and NDVI and deformation rate.

RMSE (mm/Year)	P1	P2	P3
SMMI (m^3^/m^3^)	39	24	26
NDVI	34	28	25

## Data Availability

The data used to support the findings of this study are available from the corresponding author upon request.
